# Epigenetic and microbiome responses to greens supplementation in obese older adults: results from a randomized crossover-controlled trial

**DOI:** 10.3389/fnut.2026.1750030

**Published:** 2026-02-04

**Authors:** Laura A. Robinson, Aidan M. Cavanah, Sarah Lennon, Madison L. Mattingly, William Van Der Pol, Kevin W. Huggins, Michael W. Greene, Michael D. Roberts, Andrew D. Frugé

**Affiliations:** 1 Department of Nutritional Science, Auburn University, Auburn, AL, United States; 2 College of Nursing, Auburn University, Auburn, AL, United States; 3 School of Kinesiology, Auburn University, Auburn, AL, United States; 4 Biomedical Informatics, The University of Alabama at Birmingham, Birmingham, AL, United States

**Keywords:** biological aging, DNA methylation age, epigenetic clocks, greens supplement, gut microbiome, nutritional intervention, randomized crossover trial

## Abstract

Aging is influenced by genetic, environmental, and lifestyle factors. Preliminary studies suggest that fruit and vegetable-based dietary supplements may reduce inflammation and oxidative stress, key factors in aging. Greens-based supplements typically contain concentrated extracts of leafy greens, fruits, vegetables, and bioactive phytochemicals, providing micronutrients and polyphenols that may influence aging-related pathways. This exploratory study evaluated the effects of a 30-day greens-based supplement on epigenetic markers of aging and metabolic health in adults aged 50–65 years with body mass index (BMI) >30 kg/m^2^, using a 60-day randomized crossover design. Participants were randomized to immediate or delayed supplementation. During the 30-day intervention period, participants consumed a daily greens supplement. Primary outcomes included peripheral blood mononuclear cell DNA methylation and epigenetic age (Horvath, PCGrimAge, AdaptAge, and DamAge). Secondary measures included clinical metabolic biomarkers, microbiome diversity, breath hydrogen and methane, body composition, actigraphy, dietary intake, and quality of life questionnaires [RAND 12 item short form questionnaire (SF-12), and 21-item Depression, Anxiety, and Stress Scale (DASS-21)]. Twenty-one participants began the protocol (65% female, mean age 58.4 ± 5.3 years, mean BMI 38.1 ± 8 kg/m^2^). Nineteen participants completed the study. Horvath clock data indicated that biological age paradoxically increased during the supplementation period, whereas newer-generation clocks (AdaptAge, DamAge) demonstrated trends toward improved outcomes. Gut microbiome alpha diversity remained stable; taxa of interest, including Bilophila (*p* = 0.037) and Desulfobacterota (*p* = 0.031) changed with supplementation. Body composition, metabolic biomarkers, dietary intake, breath gases, sleep, and psychosocial measures were unchanged during the study. Exploratory pre-to-post supplementation change score correlations found no significant associations between epigenetic clocks and secondary outcomes, except for an inverse relationship between Faith's phylogenetic diversity and fasting blood glucose (*r*_s_ = −0.81, *p* < 0.001). In summary, 30 days of greens-based supplementation led to selective changes in epigenetic aging markers and individual gut microbial taxa, without significant effects on overall microbiome diversity, metabolic health markers, or body composition. Additionally, exploratory correlations suggest potential links between changes in microbial diversity and glycemic control following greens supplementation.

## 1 Introduction

Biological aging is marked by progressive molecular alterations, which contribute to functional decline, greater disease susceptibility, and increased mortality risk (1–3). This multifactorial process is shaped by genetics, lifestyle, and environmental factors (2). While chronological age provides a simple measure, molecular markers such as DNA methylation patterns offer more precise indicators of individual biological aging (4, 5). The epigenome, particularly DNA methylation, provides a robust framework for measuring biological age through “epigenetic clocks” (6–8). These clocks leverage predictable methylation changes to estimate biological age and are widely used as biomarkers in aging research (6, 7). These systems represent promising targets for nutritional interventions aimed at delaying aging and preserving physiological function (8).

Diet is a powerful modulator of the hallmarks of aging, influencing pathways linked to longevity and disease risk (9). Among lifestyle factors, dietary intake is particularly accessible and modifiable (10). Nutritional interventions can alter epigenetic regulation and gut microbiome composition, both of which contribute to the progression of aging and age-associated diseases (9–11). Despite increased life expectancy, healthspan, the period lived in good health, has not risen proportionally, leaving many individuals with more years spent in poor health (12–14). Overweightness and obesity further accelerate aging through systemic inflammation, metabolic dysfunction, and heightened risk of chronic disease (12). Current approaches often intervene after physiological decline has occurred, underscoring the need for preventive strategies that target biological aging earlier in life (14, 15). Dietary intake can directly modify the epigenome by influencing methylation and gene expression without altering DNA sequence (7, 8). The gut microbiome, profoundly shaped by diet, regulates host metabolism and immune function and may also contribute to epigenetic regulation (9). Alterations in these systems can accelerate biological aging, manifesting as increased visceral adiposity, systemic inflammation, and reduced physical activity (12, 14). Targeted nutritional strategies therefore hold potential to modulate biological aging and improve healthspan (9, 10).

Building on this construct, specific nutrients and phytochemicals have been shown to directly influence DNA methylation and related epigenetic mechanisms, providing a biological basis for dietary interventions in aging research (16, 17). Greens-based supplements, often composed of leafy greens, algae (e.g., *Chlorella vulgaris, Spirulina platensis*), grasses, and concentrated plant extracts, are rich sources of these bioactives (16, 17). These supplement ingredients oftentimes supply folate, magnesium, and antioxidants that support one-carbon metabolism and DNA methylation homeostasis (16), as well as polyphenols, flavonoids, and carotenoids that can modulate DNA methyltransferases and histone-modifying enzymes (16, 18). Observational studies link diets high in leafy greens and plant bioactives with slower epigenetic aging (19, 20), while randomized trials demonstrate that algal ingredients such as *Chlorella vulgaris* reduce total cholesterol and LDL-C, and *Spirulina platensis* lower oxidative stress and inflammation (18, 21). Together, these findings suggest that a multi-ingredient/greens-based supplement may act to strategically modify DNA methylation and while also improving other physiological outcomes.

Despite growing interest in nutritional strategies to modulate biological aging, few studies have evaluated greens-based dietary supplementation using a randomized crossover design that integrates epigenetic aging markers with gut microbiome outcomes in older adults with obesity. Importantly, epigenetic clocks differ in the biological processes they are designed to capture. While first generation clocks such as Horvath primarily estimate chronological age, newer-generation clocks quantify domain-specific features of biological aging, including damage accumulation and stress responsiveness, and may therefore respond differently to short-term interventions (6–8). The present exploratory study evaluated whether a greens-based supplement consumed over 30 days could influence epigenetic markers of aging in peripheral blood mononuclear cells (PBMCs) in overweight older adults. We hypothesized that greens supplementation would favorably impact several epigenetic aging trajectories, with directionally beneficial effects on newer-generation epigenetic clocks, and that these molecular changes would occur independently of major alterations in lifestyle behaviors or body composition.

## 2 Materials and methods

### 2.1 Study design

**Figure 1:**
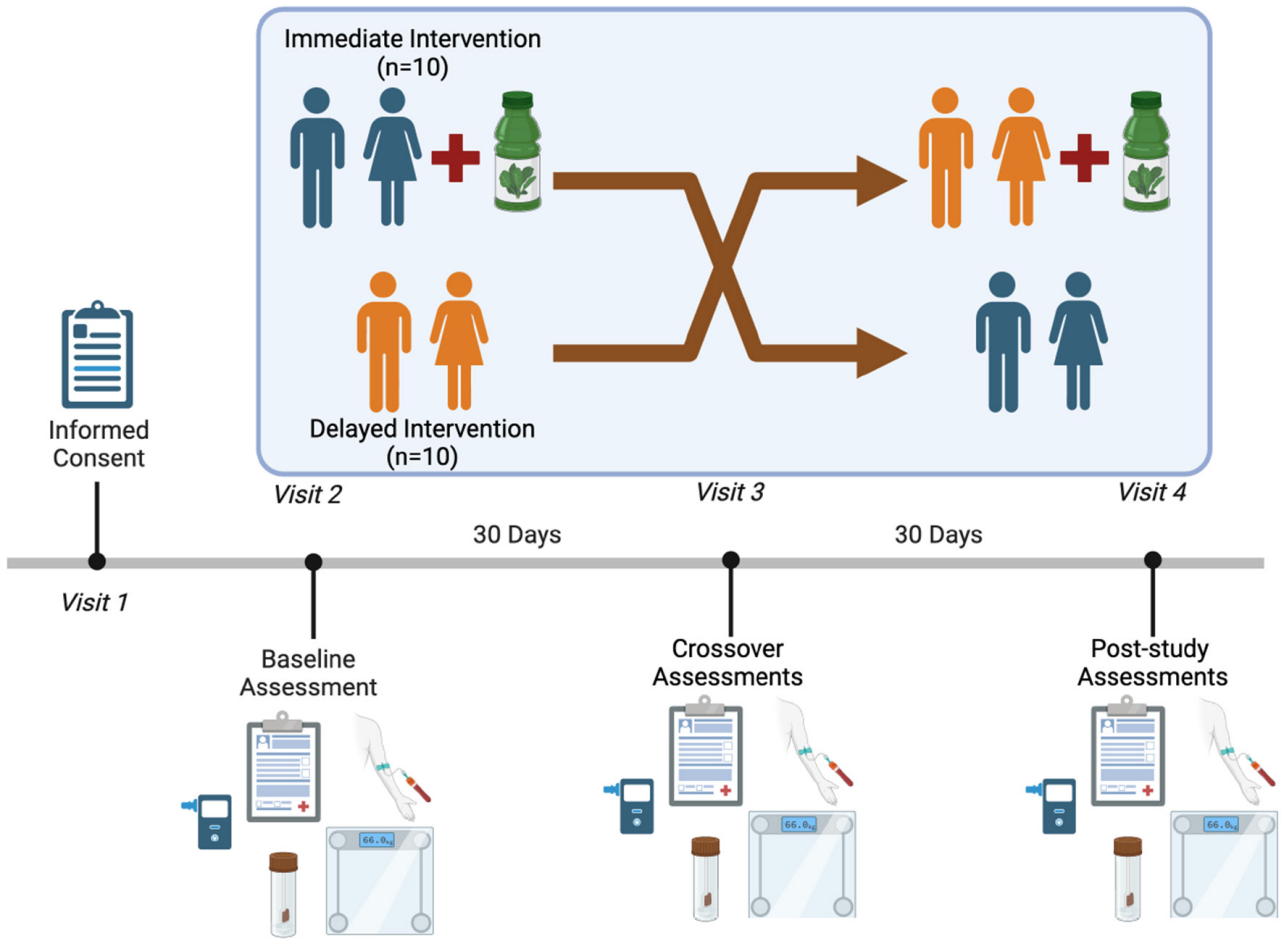
Study design.

This 60-day randomized, crossover-controlled trial was designed to evaluate the effects of a greens-based supplement on epigenetic aging and physiological outcomes in adults with obesity. Body composition, dietary intake, and physical activity were assessed as contextual measures to characterize the study population and help isolate the effects of the intervention. Twenty adults aged 50–65 years with a body mass index (BMI) > 30 kg/m^2^ were randomized to immediate or delayed intervention group (see Figure 1). The study was conducted at Auburn University School of Kinesiology Building and consisted of four total visits. Prior to data collection, this study was approved by the Auburn University Institutional Review Board (IRB; Protocol # STUDY00000013), conformed to standards set by the latest revision of the Declaration of Helsinki, and was pre-registered as a clinical trial (NCT06537232).

During Visit 1 (screening), participants provided written informed consent, completed a medical history questionnaire, and received instructions on study procedures. Eligible participants were randomized using a stratification scheme based on age (50–57 vs. 58–65 years) and race/ethnicity (non-Hispanic White vs. Other), with randomization lists generated using the Sealed Envelope Ltd Platform in blocks of four (22). Participants were randomly assigned to one of two sequences: immediate supplementation followed by a free-living phase, or vice versa. Each participant received three stool collection kits with written instructions for home use.

Visit 2 (Day 0) included baseline body composition measurements using a Tanita bioelectrical impedance analyzer Model BC-568 (Tanita Corporation of American, Inc, Arlington Heights, IL), height measurement, fasting blood collection, and breath testing for hydrogen and methane. Participants also completed the DASS-21, International Physical Activity Questionnaire (IPAQ), SF-12 Health Survey, a 24-h dietary recall, and gastrointestinal symptom visual analog scales. Stool samples, collected 24–48 h prior, were submitted during this visit. Participants issued a Garmin Vivofit 4 activity tracker (Garmin International, Olathe, KS), to wear continuously, with data uploaded to Fitabase data collection software (Fitabase, San Diego, CA).

At Visit 3 (Day 30; crossover) participants switched conditions (supplementation or free-living) and repeating all procedures performed at Visit 2, including submission of a second stool sample. Visit 4 (Day 60; endpoint) included final assessments identical to those at Visits 2 and 3, along with submission of the third and final stool sample. Adherence and symptom monitoring were monitored using a HIPPA-compliant text messaging platform, Emitrr (Emitrr Inc., https://emitrr.com). Participants received daily reminders to take the supplement during the intervention phase and were encouraged to report any adverse symptoms or missed doses via the platform. Participants experiencing significant GI discomfort were advised to discontinue the supplement and could withdraw.

### 2.2 Participant eligibility and recruitment

Sample size was determined *a priori* to detect a mean difference of 1.5 years in epigenetic age, based on prior nutritional supplementation trials. A recent study calculated that 76 participants would be needed to detect a 1.5-year change in inflammatory age (iAGE) with 80% power and a significance level of 0.05, using a paired design (23). For the present study, a power analysis using G^*^Power (version 3.1.9.7) indicated that 10 participants would provide 80% power to detect a 1.5-year within-subject difference (SD = 1.5 years, *d* = 1.0, α = 0.05). To increase statistical power while also accounting for attrition, 20 participants were enrolled.

Recruitment was conducted in the Auburn and Opelika, AL area through a Qualtrics screening survey. Recruitment was conducted over a one-month period between September and October 2024. Flyers were distributed at community locations (e.g., grocery stores, retail shops, post offices) and across Auburn University departments. The Qualtrics-hosted screening survey collected demographic and health-related information, including age, biological sex, ethnicity, height, weight, highest level of education, and marital status. Additional questions assessed the frequency and quantity of dark green leafy vegetable consumption and current use of antibiotics, immunosuppressants, corticosteroids, or probiotics. Eligible respondents were contacted and invited for an in-person screening visit, where study procedures were explained and informed consent obtained prior to participation.

### 2.3 Intervention

The greens-based supplement used in this study was a commercially available powdered blend, Field of Greens (BrickHouse Nutrition, Cooper City, FL), composed primarily of dehydrated vegetables, fruits, and botanical extracts (24). Each serving (10.43 grams) contained a proprietary mixture of organic greens including kale, spinach, parsley, chlorella, barley grass, and wheatgrass, alongside functional plant compounds such as beet root, green tea extract, turmeric, and ashwagandha. The supplement also provided B-complex vitamins (B1, B2, B6, B12, and folate), vitamin C, vitamin D3, and vitamin K2, and contained natural flavors and stevia with no added sugars. Participants were instructed to consume one full scoop mixed with water or a beverage of choice each morning during the 30-day intervention period. To maintain consistency, participants were asked to avoid introducing any new supplements, herbal products, or dietary changes for the duration of the study. Notably, this study was open-label, and no placebo supplement was consumed given the potential influence that commonly used ingredients (e.g., maltodextrin, inulin, or cellulose) could potentially affect some of the secondary outcomes (e.g., gut microbial diversity) (25).

### 2.4 Phlebotomy

At each blood draw, 7 ml of whole blood was collected into a BD Vacutainer^®^ CPT™ Mononuclear Cell Preparation Tube (Becton, Dickinson and Company, Franklin Lakes, NJ, USA) from the antecubital vein of the arm using aseptic techniques by study staff with phlebotomy training. Tubes were gently inverted after collection and centrifuged within 2 h at 1,500–1,800 RCF for ≥20 min at 18–25 °C. Peripheral blood mononuclear cells (PBMCs) and plasma were separated, aliquoted into 1 ml cryovials, and stored at−80 °C. PBMCs were shipped to TruDiagnostic (Lexington, KY, USA) for DNA methylation profiling and epigenetic clock analysis (Horvath, PCGrimAge, AdaptAge, and DamAge). Plasma samples were transported on ice to East Alabama Health Medical Center (Opelika, AL, USA) and analyzed for a comprehensive metabolic panel (CMP), triglycerides, blood glucose, and lipoproteins as we have described previously (26).

### 2.5 DNA methylation processing and clock analysis

Isolated PBMC samples were shipped to TruDiagnostics on dry ice (Lexington, KY, USA), where DNA methylation profiling was conducted using the Illumina Infinium MethylationEPIC BeadChip (>850,000 CpG sites). TruDiagnostics provided quality-controlled methylation data and epigenetic clock outputs for each participant and timepoint. To evaluate intervention effects, within-subject differences in clock scores were calculated by comparing pre- and post-intervention values for each treatment phase (supplement vs. control) according to group assignment. These difference scores were then analyzed using repeated-measures and correlational approaches to assess treatment effects and individual-level associations.

### 2.6 Epigenetic clock selection and rationale

Four established epigenetic clocks: Horvath, PCGrimAge, AdaptAge, and DamAge. These clocks were selected based on their distinct yet complementary capacities to quantify biological aging processes. The Horvath clock (353 CpG sites) estimates chronological age across tissues and remains a standard measure of biological age acceleration, with higher values indicating more advanced biological aging relative to chronological age and lower values indicating slower aging trajectories (6, 8). PCGrimAge (1,030 CpG sites) incorporates DNA methylation surrogates for plasma proteins and smoking history to predict morbidity and mortality risk, with higher values indicating elevated mortality risk and lower values generally reflecting improved healthspan (27). AdaptAge (1,000 CpG sites) is designed to quantify the biological response to environmental and physiological stress, capturing adaptive resilience mechanisms rather than cumulative damage (28). Directional changes in AdaptAge should therefore be interpreted as shifts in stress-responsive biological domains rather than strictly as changes in chronological aging; decreases may reflect improved stress adaptation capacity, whereas increases may indicate heightened stress burden or reduced resilience. DamAge (1,090 CpG sites) captures the accumulation of irreversible cellular and molecular damage associated with long-term aging burden, with higher values reflecting greater accumulation of methylation signatures linked to damage processes and lower values indicating reduced damage burden (28, 29). These clocks were selected based on evidence that aging is a modular process, with each clock representing distinct biological domains responsive to lifestyle or nutritional interventions (29, 30).

### 2.7 Body composition

Body composition was assessed at Visits 2, 3, and 4 using a multi-frequency bioelectrical impedance analyzer (Tanita BC-568; Tanita Corporation of America, Arlington Heights, IL, USA). Participants arrived fasted for ≥8 h and well hydrated. Height was measured with a stadiometer, and participants removed shoes and socks before standing on metal footplates while holding hand electrodes. The device provided estimates of body weight, body fat percentage, lean mass, and bone mass. Standardized protocols were used across visits to ensure reliability.

### 2.8 Breath gases

Breath samples were collected in duplicate at 0, 30, 60, and 90 min during Visits 2, 3, and 4 using the QuinTron EasySampler collection kit and BreathTracker SC Analyzer (QuinTron, Milwaukee, WI, USA). The greens-based supplement was consumed immediately prior to the 0-min sample during the supplementation phase (Visit 2 for the immediate group, Visit 4 for the delayed group, and Visit 3 for both groups). Participants also collected duplicate home breath samples on Days 21 and 51, timed to correspond with supplementation phases. All participants were instructed to fast ≥2 h before sampling and received reminders 2 days prior and the morning of each collection. Hydrogen, methane, and carbon dioxide were quantified, with correction factors applied by the analyzer.

### 2.9 Fecal microbial analysis

Stool samples were collected 24–48 h prior to Visits 2, 3, and 4 using OMNIgene^®^ GUT OMR-200 tubes (DNA Genotek, Ottawa, Canada) and returned at study visits. Participants recorded stool consistency using the Bristol Stool Chart and noted date and time of collection. Upon receipt, samples were stored at−80 °C until processing. Microbial DNA was extracted with Zymo Fecal/Soil DNA Miniprep kits (Irvine, CA, USA) according to manufacturer instructions. The V4 region of the 16S rRNA gene was amplified and sequenced on an Illumina MiSeq platform. Raw reads were quality-checked with FASTQC and processed in QIIME2, where DADA2 was applied for filtering, denoising, chimera removal, and amplicon sequence variant (ASV) generation (31–34). Taxonomic classification was performed against the SILVA reference database (35). Alpha diversity was calculated using observed species richness, Shannon index, and Faith's phylogenetic diversity, while beta diversity was assessed using Bray–Curtis dissimilarity and UniFrac clustering. Differential abundance testing was performed using ANCOM and DESeq2 to identify microbial taxa significantly associated with the intervention (36).

### 2.10 Activity and sleep tracking

Participants were provided with Garmin Vivofit 4 devices (Garmin International, Olathe, KS, USA) at Visit 2 and instructed to wear them continuously for the 60-day study. Study staff assisted participants with linking the devices to their smartphones, which were synced weekly through Fitabase (Small Steps Lab, LLC, San Diego, CA, USA). Daily step counts and sleep duration were extracted for analysis. Text reminders were used to encourage compliance and device syncing. Sleep phase data generated by the manufacturer were not analyzed, as the device contained only accelerometer-based sensors.

### 2.11 Subjective data

At Visits 2, 3, and 4, participants completed validated surveys to assess quality of life, psychological symptoms, activity, and gastrointestinal outcomes. The 12-Item Short Form Survey (SF-12) measured health-related quality of life across physical and mental domains (37). Depression, anxiety, and stress were assessed with the 21-item DASS-21 using a 4-point Likert scale (38). Physical activity was captured with the International Physical Activity Questionnaire, Short Form (IPAQ-SF), which was converted to MET-minutes per week (39). Gastrointestinal symptoms were assessed using a 100-mm Visual Analog Scale (GI-VAS). All questionnaires were administered via Qualtrics.

Dietary intake was assessed using three interviewer-administered 24-h recalls at each study visit, including two weekdays and one weekend day. Interviews followed the USDA multiple-pass method and included details on portion size, timing, and preparation. Recalls were analyzed in the Nutrition Data System for Research (NDSR 2024; University of Minnesota), and average daily intake was calculated for energy, macronutrients, sugars, and fiber.

### 2.12 Statistical analysis

All statistical analyses were conducted using RStudio (2025.05.0) with graphs plotted using GraphPad Prism (Version 10.1; San Diego, CA, USA). Primary outcomes included DNA methylation based epigenetic clock measures, which were evaluated using one-way repeated measures ANOVA to assess within-subject differences over time. Secondary outcomes included immune markers, breath gases, body composition, microbiome alpha diversity, actigraphy data, and scores from the SF-12 and DASS-21 questionnaires, which were analyzed using the same repeated measures framework. ANOVAs were conducted using the ezANOVA function from the ez package, with Greenhouse–Geisser corrections applied when assumptions of sphericity were violated. *Post hoc* pairwise comparisons were performed using the emmeans package with *p*-values adjusted for multiple comparisons.

Exploratory analyses included Spearman correlation coefficients with false discovery rate (FDR) correction to examine associations between epigenetic clock outcomes and physiological, microbiome, behavioral, or psychological measures across the treatment period. Categorical variables, including demographic characteristics (e.g., sex, race/ethnicity, education level) and Bristol stool types, were analyzed using Pearson's chi-square tests with Sidak adjustments. Statistical significance was set at α ≤ 0.05 for all analyses, and data are presented as means ± standard deviation values throughout.

## 3 Results

### 3.1 Study flow and participant characteristics

**Figure 2:**
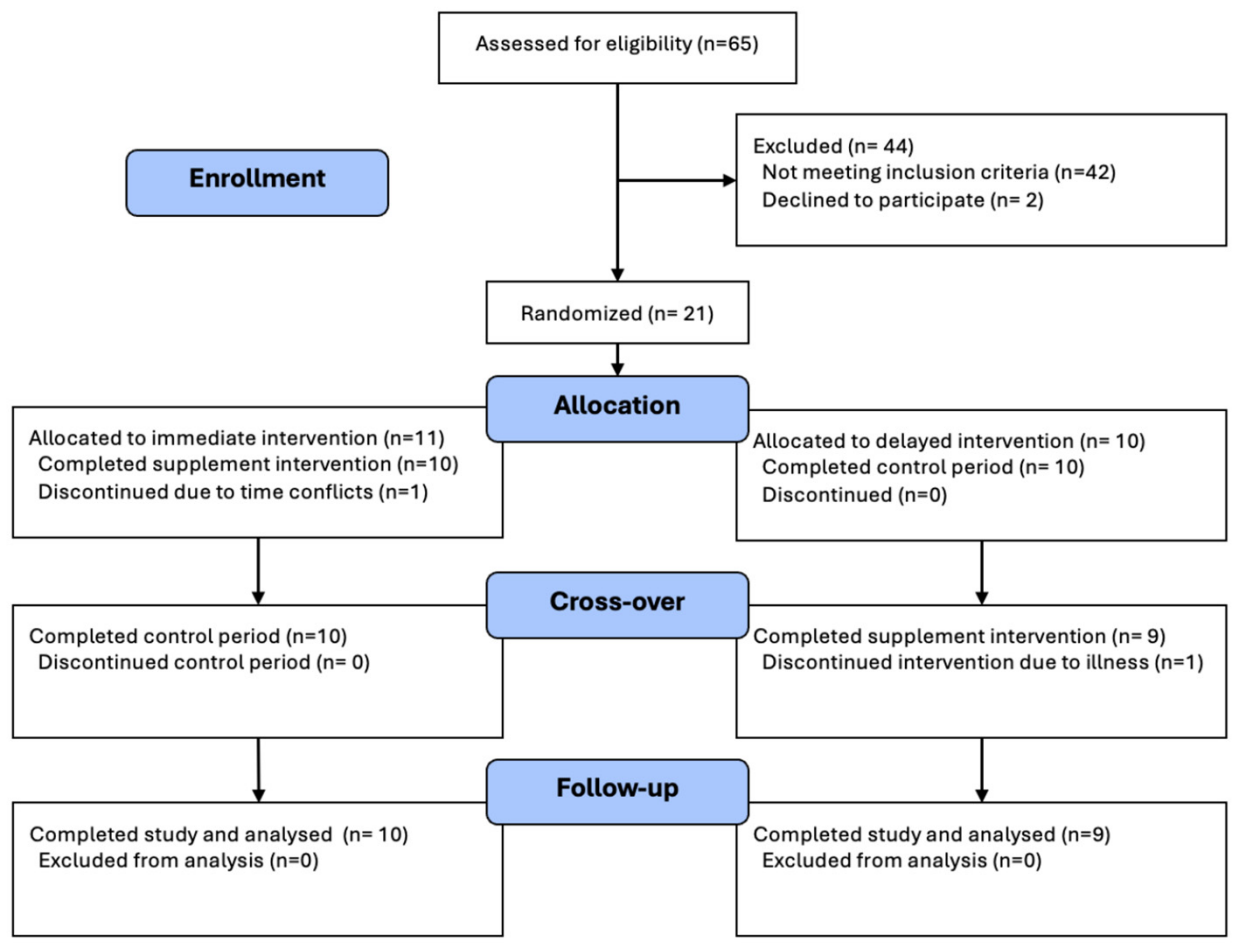
Consort flow diagram.

The CONSORT diagram (Figure 2) outlines participant accrual and retention throughout the study. Of the 65 individuals assessed for eligibility, 44 were excluded, with 42 not meeting inclusion criteria and two declining to participate. Twenty-one participants were randomized into immediate (*n* = 11) and delayed (*n* = 10) intervention groups. Two participants withdrew, one due to time conflicts, and one participant due to illness. In total, 19 participants completed the full protocol and were included in the final analyses (immediate group: *n* = 10; delayed group: *n* = 9).

Baseline demographics and characteristics are presented in Table 1. No between-group differences were observed for age (Immediate: *M* = 58.0 ± 5.0 years; Delayed: *M* = 55.0 ± 4.3 years), sex (65% female), or ethnicity. Educational attainment differed significantly (*p* = 0.044), with more college-educated participants in the immediate group. Additionally, BMI classification differed (*p* = 0.019), with more delayed group participants classified as obese class III (BMI > 40 kg/m^2^; Immediate: *M* = 34.8 ± 3.4 kg/m^2^; Delayed: *M* = 41.4 ± 9.9 kg/m^2^). Weekly servings of dark green leafy vegetables also varied slightly between groups (Immediate: *M* = 1.35 ± 1.43 servings; Delayed: *M* = 2.04 ± 1.64 servings), though this difference was not statistically significant.

**Table 1:** Baseline characteristics of study participants (*n* = 20).

**Characteristic**	**Classification**	**Total**	**Immediate *N* (%)**	**Delayed *N* (%)**	***p*-value**
Sex	Female	14 (70)	7 (70)	7 (70)	1.000
	Male	6 (30)	3 (30)	3 (30)	
Marital status	Single	2 (10)	1 (10)	1 (10)	0.934
	Married	15 (75)	8 (80)	7 (70)	
	Divorced	2 (10)	0 (0)	2 (20)	
	Widowed	1 (5)	1 (10)	0 (0)	
Ethnicity	White or Caucasian	12 (60)	6 (60)	6 (60)	0.556
	Black or African American	5 (25)	3 (30)	2 (20)	
	Hispanic, Latino, or of Spanish origin	2 (10)	1 (10)	1(10)	
	More than one race	1 (5)	0 (0)	1(10)	
Education	High school degree or equivalent (e.g. GED)	1 (5)	0 (0)	1	0.044
	Some college, no degree	2 (5)	0 (0)	2 (20)	
	Associate degree (e.g. AA, AS)	1 (5)	1 (10)	0 (0)	
	Bachelor's degree (e.g. BA, BS)	11 (55)	7 (70)	4 (40)	
	Master's degree (e.g. MA, MS, MEd)	4 (20)	1 (10)	3 (30)	
	Professional degree (e.g. MD, DDS, DVM)	1 (5)	0 (0)	1(10)	
Age (years)	mean (SD)	57 (5)	58 (5)	55 (4)	0.556
	50-57	10 (50)	4 (40)	6 (60)	
	58-65	10 (50)	6 (60)	4 (40)	
BMI (kg/m^2^)	mean (SD)	38.1 (8.0)	34.8 (3.4)	41.4 (9.9)	0.019
	30-35	10 (50)	5 (50)	5 (50)	
	36-40	4 (20)	3 (30)	1 (10)	
	>40	6 (30)	1 (10)	5 (50)	
Servings of DGLV/wk	Mean (SD)	1.7 (1.5)	1.4 (1.4)	2.0 (1.6)	0.390
	0-2	14 (70)	9 (90)	5 (50)	
	2-6	6 (30)	1 (10)	5 (50)	

Values are presented as means with standard deviation (SD) values being presented in parentheses.

BMI, body mass index; DGLV, dark green leafy vegetables.

### 3.2 Epigenetic measures

Fifteen participants (immediate group: *n* = 8; delayed group: *n* = 7) had viable PBMC samples used to assess epigenetic age across three timepoints. Participants without viable PBMC samples did not differ meaningfully from those included with respect to age, sex, BMI, or baseline clinical characteristics. As shown in Supplemental Table 2, DNA methylation age estimates, Horvath, PCGrimAge, AdaptAge, and DamAge, remained relatively stable over the 60-day study period, with no statistically significant main effects of group, time interactions. AdaptAge displayed greater variability than the other clocks, particularly in the delayed group, but the Group × Time interaction did not reach significance (*p* = 0.096).

**Figure 3:**
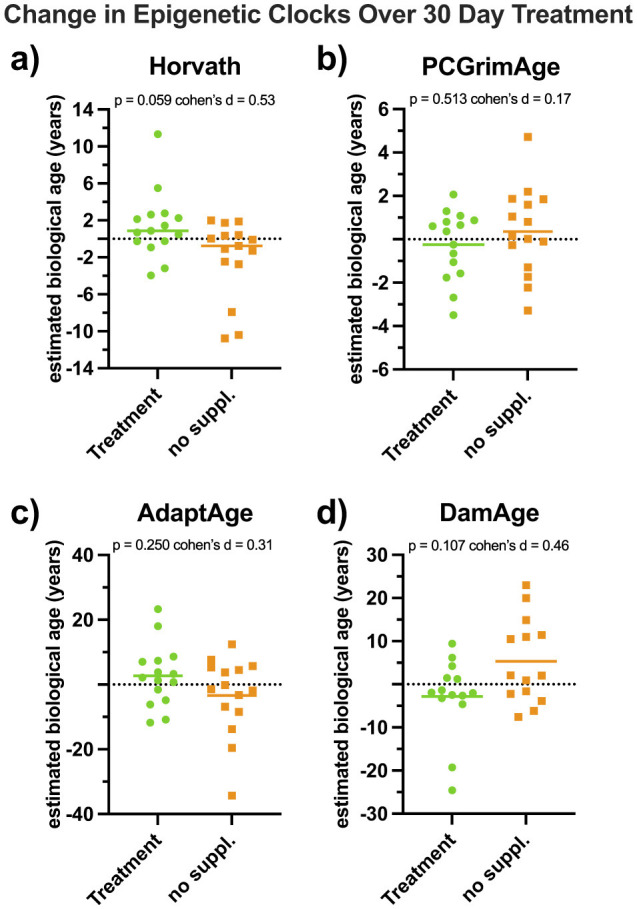
Epigenetic clock change scores. Data represent change scores in PBMC Horvath (*n* = 15, **panel a**), PCGrimAge (*n* = 15, **panel b**), AdaptAge (*n* = 15, **panel c**), and DamAge (*n* = 14, **panel d**), over the 30-day supplementation and control periods for each participant Values represent post–pre differences calculated separately for each intervention phase (supplementation vs. control) for each participant. Positive values indicate an increase in epigenetic clock estimates during the intervention period, whereas negative values indicate a decrease. Points represent individual participants; dashed lines indicate zero change. Corresponding *p*-values reflect paired (dependent samples) *t*-tests comparing supplementation and control conditions.

Figure 3 illustrates individual changes in each epigenetic clock during 30-day supplementation versus control. Horvath epigenetic age (Figure 3a) decreased during the control period and an increase during supplementation, with paired *t*-test result approaching statistical significance (*p* = 0.059). PCGrimAge, AdaptAge, and DamAge (Figures 3b–d) remained consistent (*p* = 0.369, *p* = 0.250, and *p* = 0.107).

Mean changes in epigenetic age across the 30-day supplementation and control (Supplemental Table 3) revealed that the Horvath clock age increased 1.43 ± 3.61 years during supplementation compared to a decrease of−2.07 ± 4.22 during control, yielding a significant main effect of treatment (*p* = 0.015). PCGrimAge decreased slightly during supplementation (−0.25 ± 1.59 years) and increased 0.35 ± 2.02 years during control, without significant effects. DamAge also decreased during supplementation (−2.84 ± 9.06 units) and increased 5.30 ± 9.77 units during control, with a significant group effect (*p* = 0.028) but non-significant treatment interactions. AdaptAge increased during supplementation by 3.70 ± 9.10 units during and decreased during control (−4.50 ± 11.60 units), though these changes were not statistically significant.

### 3.3 Comprehensive metabolic panel

Plasma from 18 participants were analyzed for CMP measures (immediate group: *n* = 10; delayed group: *n* = 8), with two excluded due to incomplete data. Supplemental Table 3 reports relatively stable estimated marginal means across the three study visits for most markers, with a few notable exceptions. Glucose levels decreased slightly over time in the immediate group (from 92.00 to 84.20 mg/dl) but remained relatively stable in the delayed group, resulting in a marginal group × time interaction (*p* = 0.090). Potassium and chloride exhibited significant main effects of time (*p* = 0.026 and *p* < 0.001, respectively), though changes were minor and not clearly linked to supplementation. ALT increased over time and was consistently higher in the delayed group, contributing to a near-significant interaction (*p* = 0.062) and a group effect trending toward significance (*p* = 0.085), while AST followed a similar pattern without significance. CRP was higher in the delayed group across timepoints (*p* = 0.042), but no time or interaction effects were observed. Lipid markers (HDL-C, LDL-C, total cholesterol, triglycerides) declined slightly over time in both groups, but none reached statistical significance (*p* > 0.07 for all effects). As shown in Supplemental Figure 1, paired comparisons confirmed that changes in CRP, ALT, AST, glucose, and lipids were small and did not differ between supplementation and control. Together, these findings indicate that the greens supplement did not produce meaningful changes in metabolic or inflammatory markers, although modest shifts in CRP, glucose, ALT, and electrolytes suggest potential physiological responses warranting further study.

### 3.4 Body composition and actigraphy

Two-way ANOVAs revealed no significant main effects or interactions for body weight or body fat percentage (all *p* > 0.16). On average, body weight increased by 0.33 ± 3.97 kg, and body fat percentage by 1.33 ± 6.62% during supplementation, though all effect sizes were small and not statistically significant.

**Figure 4:**
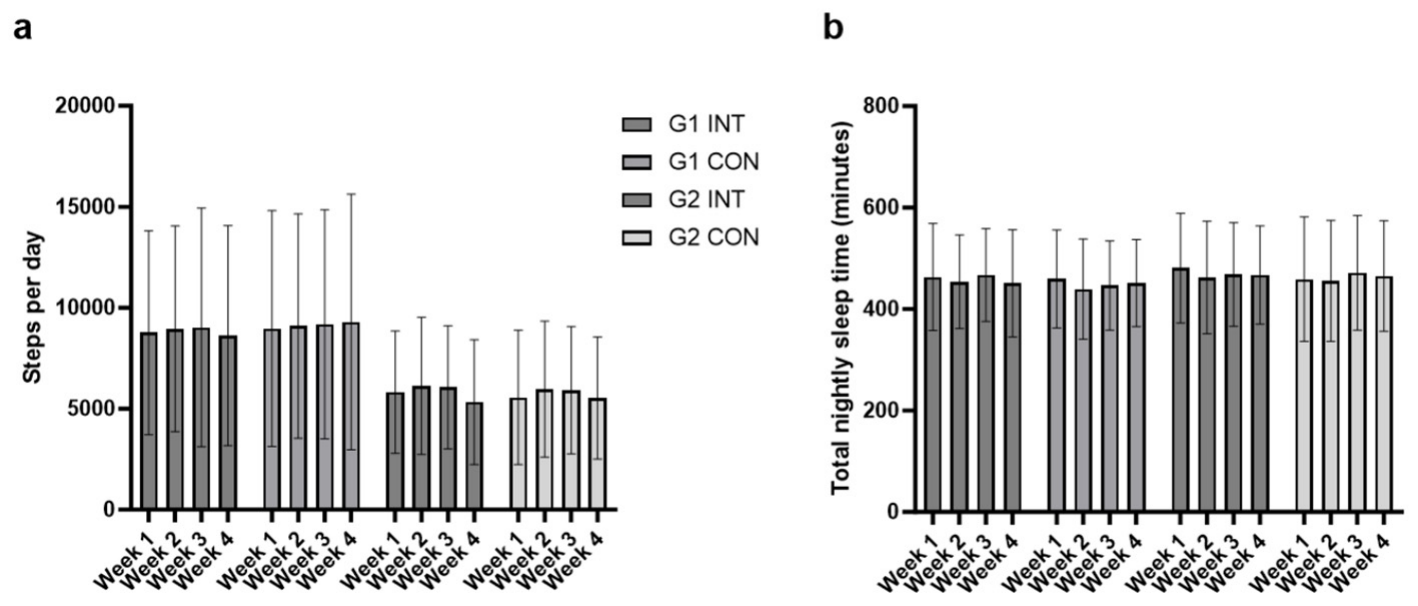
Actigraphy data. These data were provided by Garmin VivoFit4 accelerometers for steps per day (**panel a**) and Total night's sleep time (in min; **panel b**) for Immediate (G1) and Delayed (G2) groups over 4-week Intervention (INT) and 4-week Control (CON) periods (*n* = 19). Values reflect observed weekly averages.

Actigraphy data were available for 17 participants. Step counts and sleep duration remained stable across the 8-week study, with no significant effects of group, treatment, or their interaction in either ANOVA or LMM models (all *p* > 0.13). The immediate group consistently recorded higher step counts across weeks (e.g., Week 8: 8283 vs. 5158 steps/day, Δ = 3126 ± 1890, *p* = 0.113), but differences did not reach significance. Average sleep duration was ~7.7 h/night in both groups, with no meaningful variation across phases. As illustrated in Figures 4a, b, step counts and sleep duration remained relatively stable across groups and phases. These consistent findings across statistical methods support the conclusion that the greens supplement did not significantly impact physical activity or sleep behavior during the intervention period. Paired *t*-tests also confirmed no within-group changes in steps or sleep time during supplementation (all *p* > 0.39).

### 3.5 Breath hydrogen and methane

Breath samples from 19 participants were analyzed for hydrogen (H_2_) and methane (CH_4_) across three visits. At baseline (visit 2), no significant group differences were observed (H_2_: Delayed = 12.7 ± 8.73 ppm, Immediate = 5.99 ± 4.01 ppm, *p* = 0.058; CH_4_: Delayed = 4.41 ± 6.47 ppm, Immediate = 3.74 ± 5.51 ppm, *p* = 0.812). Two-way repeated measures ANOVAs showed no significant main effects of group, visit, or their interaction for either H_2_ (all *p* > 0.20) or CH_4_ (all *p* > 0.29). Although Mauchly's test indicated sphericity violations, Greenhouse-Geisser corrections did not alter the non-significant results. Short-term gas production at four timepoints (T0, T30, T60, and T90 minutes), also showed no consistent phase-dependent trends. As illustrated in Supplemental Figure 2, H_2_ ranged from 8.1 to 14.8 ppm and CH_4_ from 2.8 to 10.4 ppm, with standard deviations often exceeding 12–20 ppm, reflecting high inter-individual variability. Overall, breath hydrogen and methane concentrations varied widely between participants but were not significantly influenced by the greens supplement. Although minor visual trends (e.g., elevated CH_4_ in the immediate group at later visits) were noted, they did not reach statistical significance, suggesting colonic and small intestinal fermentation remained stable throughout the intervention.

### 3.6 Dietary intake

Nineteen participants with valid 24-h recalls across all visits were included in the analysis. Supplemental Table 4 summarizes mean intakes of energy, fat, carbohydrates, protein, cholesterol, fiber, and sugars, across the three study visits for both the immediate and delayed groups. Overall, dietary intake patterns remained stable between groups and across timepoints, with only minor numerical fluctuations. Carbohydrate intake also remained stable (all *p* > 0.20) across visits. Protein intake varied modestly across time (*p* = 0.049) but showed no group (*p* = 0.467) or interaction (*p* = 0.320) effects. Cholesterol (all *p* > 0.19), fiber (all *p* > 0.13), and sugar (all *p* > 0.40) intake did not differ significantly. Together, these findings indicate that dietary intake remained largely unchanged throughout the study, aside from a modest but statistically significant variation in protein intake over time.

### 3.7 Microbiome

**Figure 5:**
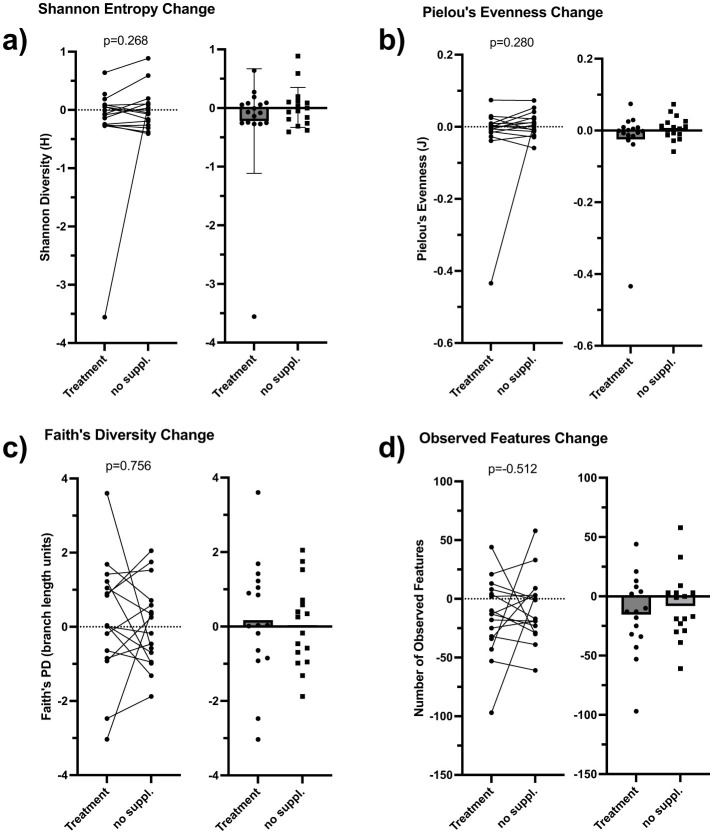
Gut microbiome alpha diversity outcomes. These data represent participant-level and group-level changes in gut microbial alpha diversity metrics including Shannon entropy (**panel a**), Faith's phylogenetic diversity (**panel b**), Pielou's evenness (**panel c**), and Observed features (**panel d**) following supplementation and control phases over 30-day period (*n* = 17). Values represent post–pre differences calculated separately for each intervention phase (supplementation vs. control) for each participant (left panels), with corresponding group-level summaries shown on the right. Positive values indicate increases in alpha diversity metrics during the intervention period, whereas negative values indicate decreases. Corresponding *p*-values represent dependent samples *t*-tests.

Seventeen participants with complete microbiome data across all visits were included in the analysis. Alpha diversity, assessed by Shannon entropy, Faith's phylogenetic diversity (PD), Pielou's evenness, and observed features, was similar between groups at baseline and remained stable throughout the study. As shown in Figure 5, individual participant statistical comparisons revealed no significant within-subject and group-level changes between supplementation and control periods (Shannon *p* = 0.268; Faith's PD *p* = 0.280; Pielou's evenness *p* = 0.756; observed features *p* = 0.521), indicating that alpha diversity remained stable. Overall microbial richness and community structure were not altered.

Beyond alpha diversity, exploratory analyses (Supplemental Table 5) showed that most phyla, including Bacteroidota, Actinobacteriota, Proteobacteria, and Firmicutes, were unaffected with supplementation. Desulfobacterota demonstrated a significant Group × Treatment interaction (*p* = 0.031), and at the genus level, Bilophila exhibited both a main effect of Treatment (*p* = 0.037) and a Group × Treatment interaction (*p* = 0.038), indicating modest, taxon-specific responses. These changes occurred in the absence of significant shifts in overall microbiome alpha diversity and therefore should be interpreted as localized microbial responses. Other genera, including Desulfovibrio, Collinsella, Bifidobacterium, Eggerthella, Adlercreutzia, Actinomyces, Escherichia-Shigella, Enterobacter, Bacteroides, and Alistipes, showed no significant effects (all *p* > 0.13). At the species level, Alistipes onderdonkii, A. putredinis, Bacteroides vulgatus, and B. fragilis did not show significant changes (all *p* > 0.08), though A. putredinis trended toward a Group × Treatment interaction (*p* = 0.081). Collectively, these results indicate that 30 days of supplementation did not significantly alter gut microbial alpha diversity or overall composition, though specific taxa such as Bilophila and Desulfobacterota showed modest, group-dependent responses.

### 3.8 Quality of life and depression scores

Nineteen participants completed all assessments of self-reported quality of life (SF-12) and psychological symptoms (DASS-21). Scores from the SF-12, including both the Physical Component (PCS) and Mental Component (MCS), remained stable across study visits, with no significant effects of group, time, or their interaction (all *p* > 0.15; Supplemental Figure 5).

Depression, anxiety, and stress scores from the DASS-21 showed no significant group differences at baseline (all *p* > 0.20) and did not change over time, with no main effects of group, time, or interaction (all *p* > 0.31; Supplemental Figure 6). Although minor baseline differences were noted, particularly for stress and depression, none reached statistical significance, and no persistent changes were observed. Overall, the greens supplement did not impact participants' quality of life or psychological distress during the study.

### 3.9 Exploratory correlations

**Figure 6:**
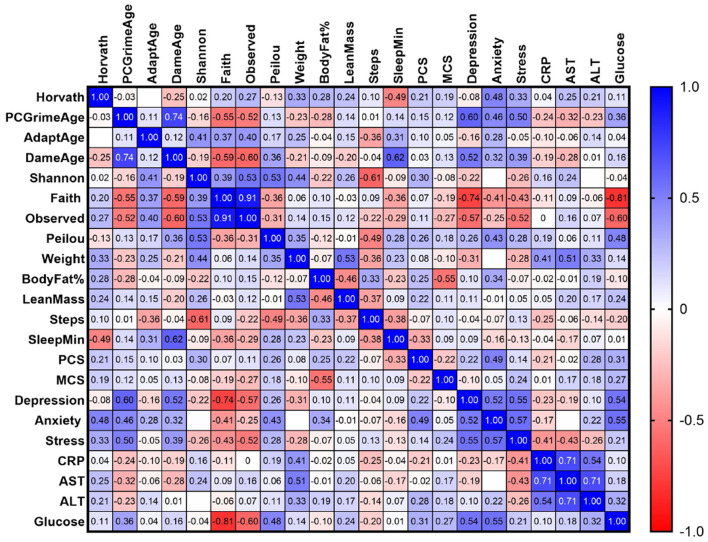
Change score correlations following greens supplementation. Exploratory Spearman correlations among within-subject pre–post change scores across epigenetic clocks, microbiome diversity indices, clinical biomarkers, body composition, actigraphy, and psychological measures following greens supplementation. Values represent Spearman correlation coefficients (*r*) calculated using individual-level change scores derived as post–pre differences for each outcome (*n* = 15). Color intensity reflects the strength and direction of associations, with blue indicating positive correlations and red indicating negative correlations. Correlations are exploratory in nature; statistical significance was assessed using false discovery rate (FDR) correction.

Correlation heatmap results (Figure 6) illustrate the strength and direction of relationships between pre-to-post green supplementation changes in epigenetic clocks, microbiome diversity indices, clinical biomarkers, body composition, and psychological outcomes. After FDR correction, no significant change score associations were observed between the primary epigenetic clocks (Horvath, AdaptAge, DamAge, and PCGrimAge) and other study variables, including each other. At the unadjusted level, Horvath age showed moderate correlations with sleep duration (*r*_s_ = −0.49, *p* = 0.069) and anxiety (*r*_s_ = 0.48, *p* = 0.070), while AdaptAge displayed weak associations with Shannon diversity, Observed species richness, and step counts (all *p* > 0.18), though none reached significance after correction. Among clinical blood biomarkers, strong positive associations were identified between AST and CRP (*r*_s_ = 0.71, *p* = 0.0006) and ALT and AST (*r*_s_ = 0.71, *p* = 0.001), with ALT also moderately associated with CRP (*r*_s_ = 0.66, *p* = 0.001). Faith's PD was inversely correlated with glucose (*r*_s_ = −0.81, *p* < 0.001), suggesting that lower microbial diversity may be linked with impaired glycemic control. Faith's PD also showed moderate inverse trends with depression and stress, though these did not persist after correction.

Microbial correlations with epigenetic clocks were further explored at multiple taxonomic levels. At the phylum level, PCGrimAge was positively associated with Proteobacteria (*r* = 0.844, *q* < 0.001), and DamAge with Bacteroidota (*r* = 0.844, *q* < 0.001). At the genus level (Supplemental Figure 3), significant associations were observed for Desulfovibrio with DamAge (*r* = 0.875, *q* < 0.001), Collinsella with PCGrimAge (*r* = 0.875, *q* < 0.001), and Eggerthella with Horvath age (*r* = 0.671, *q* = 0.024). Additional trend-level correlations included Enterobacter with Horvath (*q* = 0.055), Alistipes with AdaptAge (*q* = 0.060), and Bacteroides with DamAge (*q* = 0.091). At the species level (Supplemental Figure 4), Alistipes putredinis correlated positively with DamAge (*r* = 0.844, *q* < 0.001), while Bacteroides fragilis was associated with PCGrimAge (*r* = 0.844, *q* < 0.001).

Together, these results indicate limited evidence for broad associations between epigenetic clocks and health measures, while highlighting robust correlations between specific microbial taxa, glycemic control, and blood biomarkers.

## 4 Discussion

This randomized, crossover-controlled trial evaluated the effects of a 30-day greens-based supplement on biological aging and related outcomes in overweight and obese adults aged 50–65 years with low vegetable intake. The intervention was assessed across multiple domains, including DNA methylation-based epigenetic clocks, clinical biomarkers, psychological measures, gut microbiome diversity, fermentation gases, and actigraphy-based sleep and activity. The primary finding was a significant increase in Horvath epigenetic age during supplementation, while other clocks (PCGrimAge, AdaptAge, and DamAge) showed non-significant but small-to-moderate changes. Secondary outcomes, including clinical biomarkers, psychological symptoms, alpha diversity metrics, breath hydrogen/methane, and actigraphy measures, remained largely unchanged. Exploratory analyses revealed correlations between epigenetic clocks, microbial diversity, and behavioral factors, suggesting potential early signals of biological responsiveness.

This study adds to the growing field of nutritional epigenetics by evaluating a commercially available, mixed greens-based supplement using a randomized crossover design that integrates epigenetic aging and gut microbiome outcomes. Unlike prior trials focused on isolated nutrients, lifestyle bundles, or single epigenetic clocks, the present work assessed multiple DNA methylation clocks capturing distinct aging domains in older adults with obesity and chronically low vegetable intake (23, 40, 41). This pragmatic intervention model reflects real world dietary supplementation practices while allowing controlled within-subject comparisons of biological aging trajectories. By pairing epigenetic clock outcomes with microbiome composition and fermentation measures, this study provides a translational framework for understanding how accessible dietary strategies may influence interconnected aging-related systems.

### 4.1 Epigenetic outcomes

Interpretation of epigenetic clock outcomes requires consideration of the distinct biological domains each clock is designed to capture. The differential responsiveness of epigenetic clocks aligns with prior trials showing clock-specific effects of nutritional and lifestyle interventions. The increase in Horvath age was unexpected given observational studies linking higher fruit and vegetable intake to lower epigenetic age acceleration (42, 43). However, first-generation clocks such as Horvath are less sensitive to short-term interventions and may reflect shifts in cell composition rather than targeted biological pathways (29). This consideration may be particularly relevant in the present cohort, which had a mean BMI of 38.1 kg/m^2^, as obesity is associated with chronic low-grade inflammation, immune cell dysregulation, and altered leukocyte composition that can influence DNA methylation patterns independent of cumulative biological aging (12, 14, 15). Previous work has demonstrated that immune cell activation, differentiation, and turnover are associated with methylation changes that may occur independently of cumulative biological aging processes, reflecting shifts in immune dynamics rather than accelerated aging (44). Such effects may be particularly relevant in short-term interventions, where transient immune or inflammatory responses could influence methylation patterns at CpG sites weighted heavily in first-generation clock algorithms. In contrast, newer generation clocks capture distinct aging related domains. Second generation clocks, including AdaptAge and DamAge, exhibited effect sizes consistent with our hypotheses, though without statistical significance. These clocks capture domains related to stress adaptation and cumulative molecular damage (30), mechanisms plausibly targeted by polyphenols and bioactive compounds in leafy greens. The modest trends observed here are consistent with models proposing that clocks represent distinct biological modules of aging (45, 46).

Comparisons with prior trials further contextualize these findings. Multicomponent interventions combining diet, activity, and stress management have demonstrated reductions in epigenetic age across multiple clocks (40, 41), while nutrient-specific trials have reported more modest or null effects (23). The 30-day, single-component design of the present study may therefore have been insufficient to elicit systemic changes. Given that epigenetic aging measures reflect cumulative biological processes, longer or repeated interventions may be required to induce stable shifts in clock trajectories (19, 47). Accordingly, the 30-day, single-component design of the present study may have captured early or adaptive responses rather than durable epigenetic remodeling. Nonetheless, the moderate effect sizes observed for AdaptAge and DamAge indicate directional trends consistent with the biological domains captured by these clocks; however, given the modest sample size and lack of statistical significance, these findings should be interpreted as exploratory rather than evidence of efficacy.

### 4.2 Clinical and physiological biomarkers

Clinical markers including glucose, ALT, AST, CRP, lipids, and electrolytes remained largely stable across intervention phases. Small fluctuations were observed, such as higher CRP and ALT in the delayed group and a modest decline in glucose in the immediate group, but none reached statistical significance. These findings suggest limited short-term physiological effects in a relatively healthy population. Exploratory correlations provided additional insight. AST was positively associated with CRP, supporting links between liver function and systemic inflammation. CRP also trended with body fat percentage but not with epigenetic clocks. These results are consistent with prior trials showing that baseline inflammatory status moderates biomarker responsiveness (20, 23). The absence of group-level changes here may reflect low baseline inflammation, short intervention duration, and reliance on supplementation rather than whole-food dietary shifts (20, 48).

### 4.3 Microbiome and fermentation activity

No significant changes were detected in microbiome alpha diversity or fermentation gases, diverging from some longer or more intensive fruit and vegetable-based interventions (49, 50). However, exploratory correlations revealed that greater microbial richness (Faith's PD, observed species) was linked to lower glucose, depression, and stress scores, although only the glucose association remained after correction. These findings align with growing evidence reporting associations between specific microbial taxa and metabolic or psychological outcomes, rather than global changes in microbiome diversity, potentially reflecting shared gut–brain and immune-related processes (51–54). Taxonomic correlations suggested that pro-inflammatory taxa, including Proteobacteria, Desulfovibrio, and Eggerthella, were positively associated with accelerated epigenetic age, whereas Bacteroides and Lachnospira were inversely associated with damage related clocks. These patterns are consistent with prior work linking microbial composition to systemic inflammation and aging processes (55–58). Importantly, the absence of changes in alpha diversity indicates that greens supplementation did not induce broad microbiome remodeling over the 30-day intervention, and observed effects should be interpreted as taxon-specific. Such results underscore the potential interplay between diet, the microbiome, and epigenetic regulation.

### 4.4 Actigraphy and self-reported outcomes

No significant group-level changes were observed in daily step counts, sleep duration, or self-reported psychological measures. Nonetheless, Horvath age showed a trend toward inverse association with sleep duration and positive association with anxiety, supporting prior studies linking poor sleep and psychological distress with accelerated epigenetic aging (59–62). AdaptAge also trended inversely with daily steps, suggesting potential sensitivity of newer clocks to behavioral inputs. While non-significant, these patterns are consistent with prior literature identifying sleep and activity as potential modifiers of biological aging but should be interpreted as exploratory associations in the context of the present study. However, given the open-label design, any subjective trends observed should be interpreted cautiously, as expectation effects may influence self-reported outcomes independent of physiological change.

### 4.5 Strength and limitations

This study has several strengths, including a randomized crossover design that minimized interindividual variability and strengthened causal inference for molecular outcomes known to exhibit high between-subject heterogeneity. Additional strengths include the integration of multiple DNA methylation based epigenetic clocks capturing distinct biological domains, the concurrent assessment of epigenetic aging and gut microbiome outcomes, and the focus on an at-risk population with chronically low vegetable intake. The use of a commercially available, mixed greens-based supplement further enhances the translational relevance of the findings by reflecting a pragmatic, real-world dietary intervention.

The absence of a placebo control and the use of an open-label design represent key limitations, introducing the potential for expectation bias, particularly for subjective and behaviorally mediated outcomes such as sleep, quality of life, and psychological measures. Participant awareness of supplementation may have influenced self-reported perceptions or behavioral engagement independent of biological effects. Other limitations include the short intervention duration, modest sample size, and lack of dietary control; in addition, compliance with the greens supplement was not objectively verified using biomarkers such as plasma carotenoids or folate, and adherence was inferred from self-reported dietary recalls rather than biochemical confirmation. Menopausal status was not formally assessed, and the inclusion of women across a transitional reproductive age range may have contributed to variability in epigenetic aging measures. The modest sample size, combined with the known inter-individual variability of DNA methylation based epigenetic clocks, limits statistical power to detect small-to-moderate effects. Accordingly, this study should be viewed as exploratory and hypothesis-generating rather than confirmatory.

Additionally, the reliance on 16S rRNA sequencing without additional shotgun metagenomic or metatranscriptomic testing limits insight into microbial functional capacity or metabolic activity. As such, interpretations related to inflammation, metabolism, or epigenetic regulation should be considered speculative and based on inferred associations rather than direct functional evidence. Lastly, the supplement-only design, without concurrent lifestyle modifications, may have restricted the magnitude of observed effects.

## Conclusions

This randomized crossover trial provides preliminary, exploratory evidence that 30 days of greens supplementation significantly increased Horvath epigenetic age, while newer-generation clocks (AdaptAge, DamAge) showed non-significant trends consistent with physiological improvements. No significant effects were observed for clinical biomarkers, microbiome diversity, actigraphy, or psychological outcomes, although exploratory correlations suggested potential interactions between microbial diversity, behavior, and epigenetic regulation. Given the modest sample size and variability inherent to epigenetic aging measures, these findings should be interpreted as hypothesis-generating, highlighting the need for larger, longer-duration, and blinded trials to clarify the role of greens-based supplementation in modulating biological aging. By integrating multiple domains of biological and behavioral health, this study aimed to provide a comprehensive assessment of the potential role of greens supplementation in modulating biological aging. Overall, the results do not provide conclusive evidence for clinical modulation of biological aging; however, the absence of adverse effects and the presence of clock-specific trends suggest that greens supplementation may hold potential biological relevance, warranting further investigation in longer and adequately powered trials.

## Data Availability

The original contributions presented in the study are publicly available. This data can be found here: https://www.ncbi.nlm.nih.gov/bioproject/PRJNA1403516.
