# Supplementary material for: Epigenetic and microbiome responses to greens supplementation in obese older adults: results from a randomized crossover-controlled trial
Source: Front Nutr. 2026 Feb 4;13:1750030. doi: 10.3389/fnut.2026.1750030 (PMC12915338; doi:10.3389/fnut.2026.1750030)
Supplement: Supplementary file 1 [file Table_1.docx]

Supplementary Material

# Supplementary Data

Supplementary Material should be uploaded separately on submission. Please include any supplementary data, figures and/or tables.

Supplementary material is not typeset so please ensure that all information is clearly presented, the appropriate caption is included in the file and not in the manuscript, and that the style conforms to the rest of the article.

## Epigenetic Measures

# Table 1. Means and Group × Time Effects for Longitudinal Changes in Epigenetic Markers (n=15)

|  | Immediate n=8 | | | Delayed n=7 | | |  |  |  |
| --- | --- | --- | --- | --- | --- | --- | --- | --- | --- |
|  | Baseline | Day 30 crossover | Day 60 | Baseline | Day 30 crossover | Day 60 | Group | Time | Group x Time |
|  | Mean (SD) | Mean (SD) | Mean (SD) | Mean (SD) | Mean (SD) | Mean (SD) | p | p | p |
| Horvath | 61.8 (5.88) | 61.7 (5.51) | 60.5 (4.83) | 62.4 (4.96) | 59.3 (8.47) | 62.5 (7.48) | 0.987 | 0.348 | 0.122 |
| PCGrimAge | 62.3 (2.69) | 61.7 (3.2) | 62.4 (2.84) | 60.6 (2.9) | 60.5 (4.77) | 60.6 (3.76) | 0.376 | 0.538 | 0.732 |
| AdaptAge | 56.6 (5.56) | 57.9 (7.06) | 51.3 (12.9) | 55 (12.4) | 55.4 (18.3) | 59.7 (11.2) | 0.791 | 0.903 | 0.096 |
| DamAge | 38.4 (7.28) | 38.2 (9.86) | 44 (12.3) | 37.5 (10.5) | 39.8 (20.6) | 35.8 (8.48) | 0.670 | 0.696 | 0.100 |

# Table 2. Change in Epigenetic Age over 30-day Supplementation and Control Periods and Associated ANOVA p-values for Group, Treatment, and Interaction Effects

|  | ∆ with 30-d Supplementation | ∆ with 30-d Control | Group | Treatment | Group x Treatment |
| --- | --- | --- | --- | --- | --- |
|  | Mean (SD) | Mean (SD) | p | p | p |
| Horvath (353 CpG sites) | 1.43 (3.61) | -2.07 (4.22) | 0.630 | 0.015* | 0.090 |
| PCGrimAge (1,030 CpG sites) | -0.25 (1.59) | 0.35 (2.02) | 0.419 | 0.895 | 0.247 |
| AdaptAge (1,000 CpG sites) | 3.70 (9.10) | -4.50 (11.60) | 0.151 | 0.226 | 0.620 |
| DamAge (1,090 CpG sites) | -2.84 (9.06) | 5.30 (9.77) | 0.028* | 0.330 | 0.515 |

#

# Legend: These show Greens versus no supplementation results for four highly regarded biological clocks (n=15 participants for Horvath, PCGrimAge, and AdaptAge, while n=14 participants for DamAge). Increases with AdaptAge are viewed as positive aging alterations and increases in the other four clocks are viewed as negative aging alterations.

## Comprehensive Metabolic Panel

Table 3. Means of Key Metabolic Biomarkers by Group Across Study Visits (n=18)

|  | Immediate n=10 | | | Delayed n=8 | | |  |  |  |
| --- | --- | --- | --- | --- | --- | --- | --- | --- | --- |
| Variable (units) | Baseline | Day 30 crossover | Day 60 | Baseline | Day 30 crossover | Day 60 | Group  p-value | Time  p-value | GxT  p-value |
| Glucose (mg/dL) | 92.0 (13.6) | 87.8 (10.8) | 84.2 (11.7) | 84.5 (10.5) | 87.0 (8.9) | 87.6 (11.4) | 0.728 | 0.623 | 0.090 |
| Sodium (mM) | 163.3 (2.9) | 163.3 (8.5) | 162.9 (9.9) | 167.0 (4.0) | 163.6 (5.40) | 160.8 (10.3) | 0.719 | 0.471 | 0.555 |
| Potassium (mM) | 3.32 (0.32) | 3.28 (0.38) | 3.14 (0.12) | 3.31 (0.30) | 3.11 (0.22) | 3.09 (0.23) | 0.467 | 0.026* | 0.516 |
| Chloride (mM) | 79.0 (4.4) | 77.1 (4.18) | 74.8 (4.9) | 80.6 (3.7) | 75.1 (3.04) | 75.8 (5.4) | 0.900 | >0.001* | 0.270 |
| ALT (U/L) | 10.50 (5.23) | 12.40 (7.06) | 13.80 (3.71) | 18.62 (6.41) | 13.50 (6.26) | 16.25 (5.68) | 0.085 | 0.366 | 0.062 |
| AST (U/L) | 15.50 (3.66) | 16.20 (3.85) | 16.90 (4.65) | 21.75 (8.36) | 18.00 (6.97) | 20.88 (9.16) | 0.124 | 0.402 | 0.301 |
| HDL-C (mg/dL) | 37.7 (8.4) | 35.0 (7.2) | 37.5 (6.4) | 38.0 (5.0) | 35.8 (9.3) | 33.3 (9.1) | 0.747 | 0.147 | 0.326 |
| LDL-C (mg/dL) | 88.7 (28.6) | 82.1 (22.8) | 87.1 (24.8) | 85.3 (18.3) | 79.5 (21.0) | 75.0 (21.7) | 0.568 | 0.145 | 0.326 |
| Total Chol. (mg/dL) | 139.2 (35.3) | 131.9 (29.9) | 134.5 (30.6) | 133.5 (20.6) | 126.5 (25.8) | 121.5 (25.1) | 0.536 | 0.132 | 0.616 |
| Triglycerides  (mg/dL) | 76.4 (29.1) | 76.5 (31.7) | 77.8 (34.9) | 100.8 (28.4) | 99.4 (29.9) | 108.5 (44.5) | 0.072 | 0.732 | 0.846 |
| CRP (mg/L) | 0.23 (0.13) | 0.21 (0.12) | 0.22 (0.11) | 0.47 (0.23) | 0.44 (0.35) | 0.38 (0.31) | 0.042* | 0.313 | 0.414 |

Table legend: Values are presented as means with standard deviation (SD) values being presented in parentheses. Abbreviations: ALT, alanine aminotransferase; AST, aspartate aminotransferase; HDL-C, high density lipoprotein cholesterol; LDL-C, low density lipoprotein cholesterol; Total Chol., total cholesterol; CRP, C-reactive protein.

Figure 1. Paired changes in CRP, ALT, AST, and glucose during control and supplementation periods for each group (n = 18)


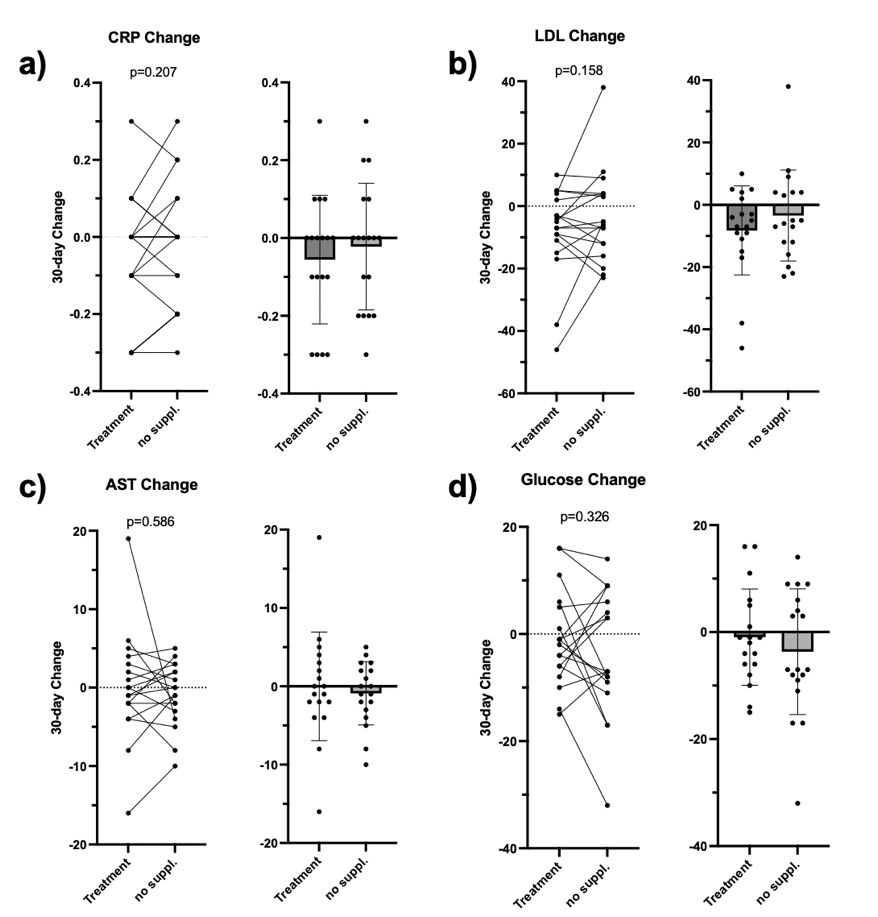


As shown in Supplemental Figure 4, changes in CRP (panel a), LDL (panel b), AST (panel c), and glucose (panel d) were visually small and did not differ significantly between groups. CRP levels were similar across conditions (p = 0.546), with a small effect size (Cohen’s *d* = 0.20) and a mean change of –0.02 mg/L during the control period versus –0.06 mg/L during supplementation. ALT showed a negligible difference (p = 0.854, *d* = –0.06), with means of –0.44 U/L and 0.00 U/L for control and supplementation, respectively. AST also did not differ significantly (p = 0.642, *d* = –0.16), with a mean change of –0.89 U/L during control and 0.00 U/L during supplementation, consistent with the variability shown in Figure 4c.

Glucose levels declined slightly more in the control period (–3.67 mg/dL) compared to supplementation (–0.94 mg/dL), though this difference was not significant (p = 0.440, *d* = –0.26), as reflected in Figure 4d. Triglyceride levels increased during supplementation (mean change = +3.50 mg/dL) and remained nearly unchanged during control (+0.17 mg/dL), but the difference was not statistically significant (p = 0.716, *d* = –0.12).

Total cholesterol showed a small, non-significant difference (p = 0.539, *d* = 0.21), with a mean change of –5.72 mg/dL in the control period and –9.39 mg/dL in supplementation. Similarly, LDL cholesterol decreased by –3.44 mg/dL in control and –8.22 mg/dL in supplementation, with a small effect size (p = 0.328, *d* = 0.33), as illustrated in Figure 4b. HDL cholesterol declined slightly more in supplementation (–3.39 mg/dL) compared to control (–1.39 mg/dL), though again the difference was non-significant (p = 0.302, *d* = 0.35).

## Breath Hydrogen and Methane

Figure 2. Individual and Group-Level Postprandial Breath Hydrogen and Methane Concentrations at T0, T30, T60, and T90 Minutes over visit 2, 3, and 4 (n = 19)


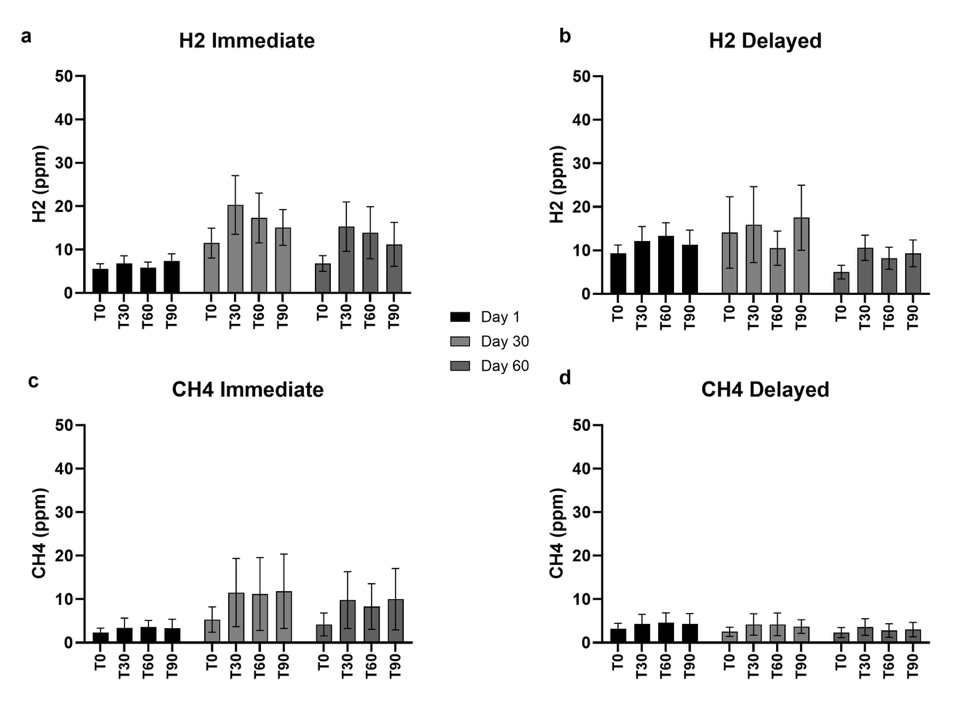


## Dietary Intake

Table 4. Self-reported Dietary Intakes at Baseline, Day 30 (Crossover), and Day 60 (Final) visits (n = 19)

|  | Immediate n=10 | | | Delayed n=9 | | |  |  |  |
| --- | --- | --- | --- | --- | --- | --- | --- | --- | --- |
| Variable (units) | Baseline | Day 30 crossover | Day 60 | Baseline | Day 30 crossover | Day 60 | Group p-value | Time p-value | G x T p-value |
| Energy (kcal/d) | 1889 (500) | 1991 (670) | 2150 (691) | 2011 (317) | 1789 (513) | 1939 (700) | 0.667 | 0.483 | 0.340 |
| Fat (g/d) | 82.1 (29.8) | 92.4 (32.4) | 99.3 (39.6) | 89.4 (14.1) | 83.6 (32.0) | 85.1 (44.3) | 0.672 | 0.69 | 0.349 |
| Carbohydrate (g/d) | 221 (52.8) | 204 (82.9) | 215 (64.3) | 223 (51.4) | 188 (64.0) | 206 (51.9) | 0.769 | 0.209 | 0.819 |
| Protein (g/d) | 72.6 (21.3) | 82.2 (21.2) | 95.9 (32.5) | 76.6 (14.1) | 67.4 (17.1) | 83.5 (50.5) | 0.467 | 0.049* | 0.320 |
| Cholesterol (mg/d) | 316 (120) | 367 (123) | 406 (207) | 302 (115) | 249 (79.5) | 321 (210) | 0.197 | 0.312 | 0.442 |
| Dietary Fiber (g/d) | 23.1 (16.6) | 19.3 (8.8) | 22.6 (12.5) | 17.9 (5.1) | 15.5 (6.3) | 13.3 (9.3) | 0.173 | 0.137 | 0.353 |
| Sugars (g/d) | 85.7 (30.4) | 78.4 (44.3) | 76.9 (36.4) | 96.5 (35.9) | 86.8 (45.1) | 85.7 (40.8) | 0.531 | 0.401 | 0.991 |

Table legend: Values are presented as means with standard deviation (SD) values being presented in parentheses.

## Microbiome

Table 5. Change in Alpha Diversity and Microbial Taxa over 30-day Supplementation and Control Periods and Associated ANOVA p-values for Group, Treatment, and Interaction Effects (n = 17)

|  |  | ∆ with 30-d Supplementation | ∆ with 30-d Control | Group | Treatment | Group x Treatment |
| --- | --- | --- | --- | --- | --- | --- |
|  |  | Mean (SD) | Mean (SD) | p | p | p |
| Alpha Diversity | Shannon Entropy | -0.033 (0.242) | 0.006 (0.353) | 0.918 | 0.796 | 0.729 |
|  | Pielou’s Evenness | 0.382 (1.411) | 0.012 (1.16) | 0.934 | 0.536 | 0.553 |
|  | Fath PD | 0.003 (0.026) | 0.007 (0.034) | 0.529 | 0.861 | 0.395 |
|  | Observed Features | -9.933 (25.877) | -8.8 (29.172) | 0.291 | 0.815 | 0.532 |
| Phyla | Bacteroidota | -917.933 (6870.909) | -2953.6 (3842.36) | 0.850 | 0.352 | 0.991 |
|  | Desulfobacterota | 1.267 (96.409) | -30.467 (104.286) | 0.660 | 0.187 | 0.031 |
|  | Actinobacteriota | 45.467 (623.973) | 147.4 (878.628) | 0.913 | 0.859 | 0.230 |
|  | Proteobacteria | -229.067 (739.43) | 189.133 (1253.52) | 0.200 | 0.355 | 0.443 |
|  | Firmicutes | -7947.733 (14406.24) | 899.467 (12943.197) | 0.141 | 0.593 | 0.352 |
| Genus | Bilophila | 10.7 (67.366) | -37.1 (84.483) | 0.037* | 0.682 | 0.038* |
|  | Desulfovibrio | -8.3 (40.197) | 6.3 (37.194) | 0.899 | 0.452 | 0.246 |
|  | Collinsella | 88.2 (495.074) | 57.4 (243.242) | 0.655 | 0.933 | 0.236 |
|  | Bifidobacterium | 12.9 (212.346) | 63.9 (756.822) | 0.937 | 0.635 | 0.428 |
|  | Eggerthella | -3.9 (25.783) | 6.5 (20.103) | 0.312 | 0.545 | 0.451 |
|  | Adlercreutzia | -9.3 (38.468) | 12.3 (24.176) | 0.133 | 0.955 | 0.271 |
|  | Actinomyces | -1.4 (6.685) | 1.8 (8.76) | 0.197 | 0.985 | 0.251 |
|  | Escherichia-Shigella | -11.4 (35.813) | 192 (853.216) | 0.350 | 0.759 | 0.333 |
|  | Enterobacter | -97.2 (422.929) | 44 (176.924) | 0.333 | 0.481 | 0.361 |
|  | Bacteroides | -1463.7 (4144.253) | -2344.6 (3616.162) | 0.438 | 0.932 | 0.348 |
|  | Alistipes | -145.9 (874.715) | 71.2 (619.703) | 0.420 | 0.447 | 0.736 |
| Species | Alistipes_onderdonkii | -38.2 (138.2789) | -7.333 (64.047) | 0.260 | 0.460 | 0.964 |
|  | Alistipes_putredinis | -107.067 (351.166) | -6.933 (356.547 | 0.578 | 0.263 | 0.081 |
|  | Bacteroides_vulgatus | -405.333 (2936.227) | -187.375 (1571.455) | 0.991 | 0.325 | 0.229 |
|  | Bacteroides_fragilis | -13.6 (234.585) | -65.533 (251.405) | 0.774 | 0.473 | 0.410 |
|  | Bilophila_wadsworthia | -0.2 (28.751) | -30.867 (76.636) | 0.096 | 0.996 | 0.147 |

Beyond alpha diversity, exploratory analyses, using two-way ANOVA, were conducted to assess microbial changes at the phylum, genus, and species levels. No significant treatment effects were observed for most phyla, including Bacteroidota, Actinobacteriota, Proteobacteria, and Firmicutes (all p > 0.14). However, a significant Group × Treatment interaction was detected for Desulfobacterota (p = 0.031), suggesting differential changes by intervention order.

At the genus level, Bilophila exhibited a significant main effect of Treatment (p = 0.037) and a significant Group × Treatment interaction (p = 0.038), indicating a potential response to supplementation that varied by group. These patterns support a potential effect of the greens supplement on specific taxa, although further studies are needed to confirm these changes across larger samples and over longer durations. No other genera, including Desulfovibrio, Collinsella, Bifidobacterium, Eggerthella, Adlercreutzia, Actinomyces, Escherichia-Shigella, Enterobacter, Bacteroides, or Alistipes, showed statistically significant differences (all p > 0.13).

At the species level, *Alistipes onderdonkii*, *Alistipes putredinis*, *Bacteroides vulgatus*, and Bacteroides fragilis did not show significant treatment or interaction effects (all p > 0.08). The closest to significance was *Alistipes putredinis*, which showed a trend-level Group × Treatment interaction (p = 0.081), potentially indicating differential species-level responses depending on intervention timing.

Figure 3. Spearman Correlation Pre-to-Post intervention Change Between Genus Taxa and Markers of Epigenetic Age (n = 15)


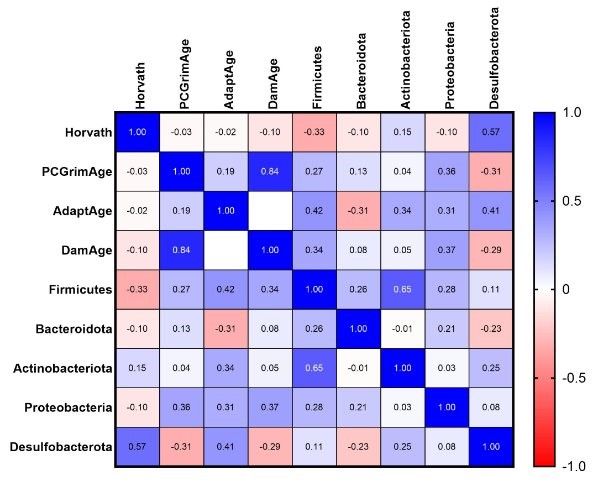


Finally, as illustrated in Figure 3, four species previously linked to aging and inflammation were examined: Alistipes putredinis, Alistipes onderdonkii, Bacteroides fragilis, and Bacteroides vulgatus. After FDR correction, two species–clock correlations were statistically significant. Alistipes putredinis was positively correlated with DamAge (r = 0.844, q < 0.001), and Bacteroides fragilis was positively associated with PCGrimAge (r = 0.844, q < 0.001). No other species showed significant associations.

Figure 4. Spearman Correlation Pre-to-Post intervention Change Between Species Taxa and Markers of Epigenetic Age (n = 15)


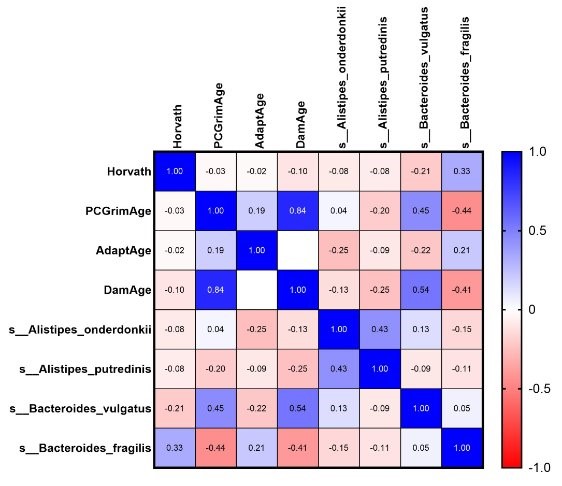


Figure 5. Individual SF-12 a) physical and b) mental component scores over entire study period (n =19)


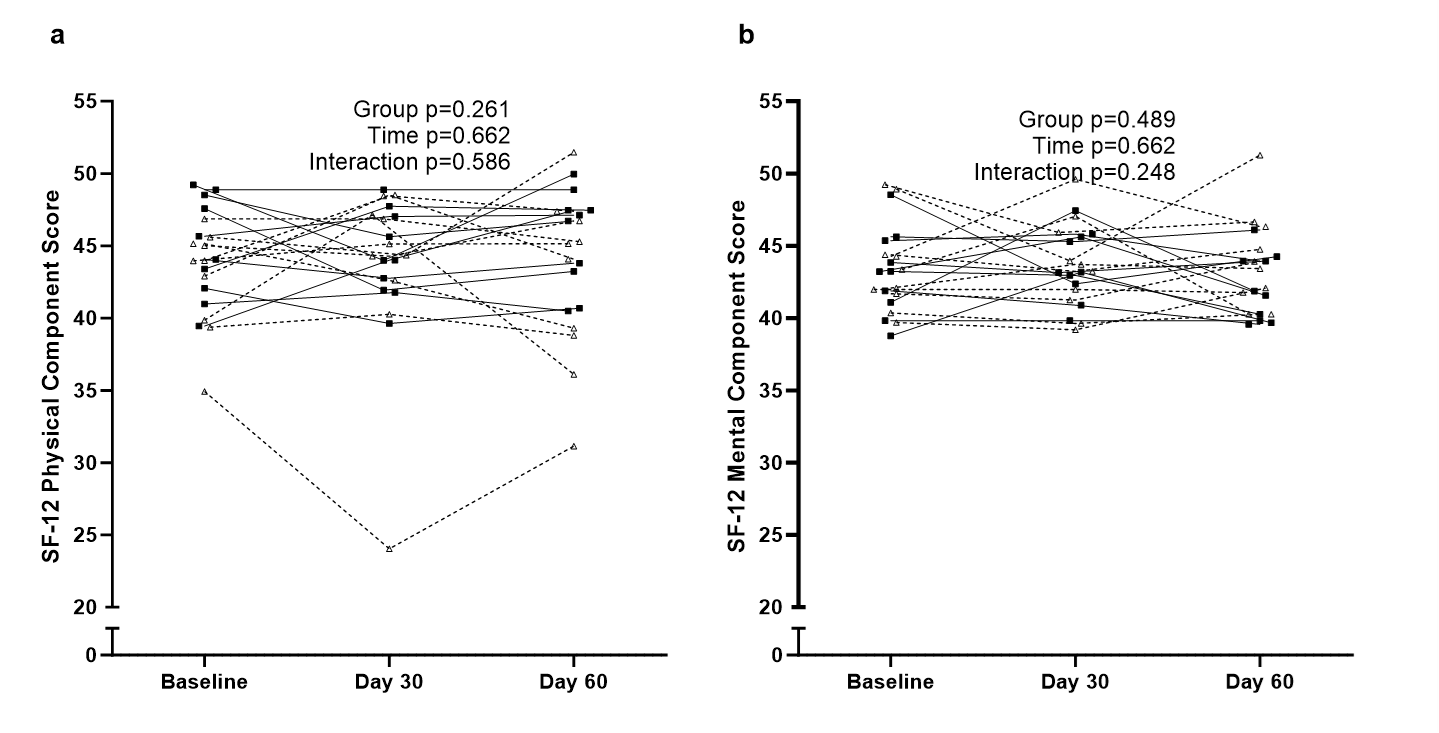


Figure 6. Individual DASS21 subscales a) Depression, b) Anxiety, and c) Stress, over entire study period (n = 19)


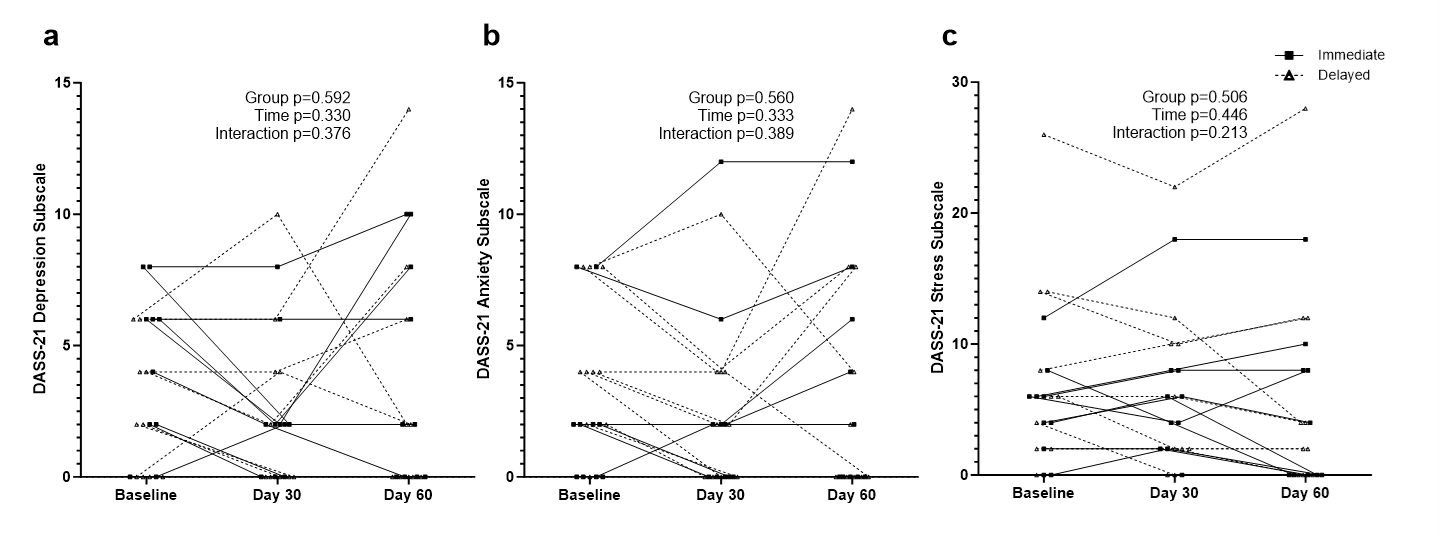


Figure 6. illustrates changes in DASS-21 subscale scores for depression, anxiety, and stress over time. At baseline, there were no significant differences between groups for depression (p = 0.293), anxiety (p = 0.251), or stress (p = 0.209). For depression (Figure 6a), no significant main effects were observed for group (F(1,17) = 0.13, p = 0.724), time (F(2,34) = 1.01, p = 0.376), or group × time interaction (F(2,34) = 1.20, p = 0.315). For anxiety (Figure 6b), no significant effects were found for group (p = 0.476), time (p = 0.393), or interaction (p = 0.539), and all within-group comparisons were non-significant. Stress scores (Figure 6c) fluctuated modestly over time but showed no significant group effect (F(1,17) = 0.77, p = 0.393), time effect (F(2,34) = 0.56, p = 0.578), or interaction (F(2,34) = 1.17, p = 0.322). LMMs for the DASS-21 subscales yielded consistent findings, with no significant effects for depression (p = 0.613), anxiety (p = 0.357), or stress (p = 0.844), and all interaction terms remained non-significant (p > 0.25).
